# Supplementary material for: The interactome of intact mitochondria by cross-linking mass spectrometry provides evidence for coexisting respiratory supercomplexes
Source: Mol Cell Proteomics. 2017 Dec 8;17(2):216–32. doi: 10.1074/mcp.RA117.000470 (PMC5795388; doi:10.1074/mcp.RA117.000470)
Supplement: Supplemental Data [file supp_RA117.000470_133922_0_supp_23978_fzffwf.zip › spectra_annotation/mito_spectra_annotation/135-1-3-1-2-1.pdf]

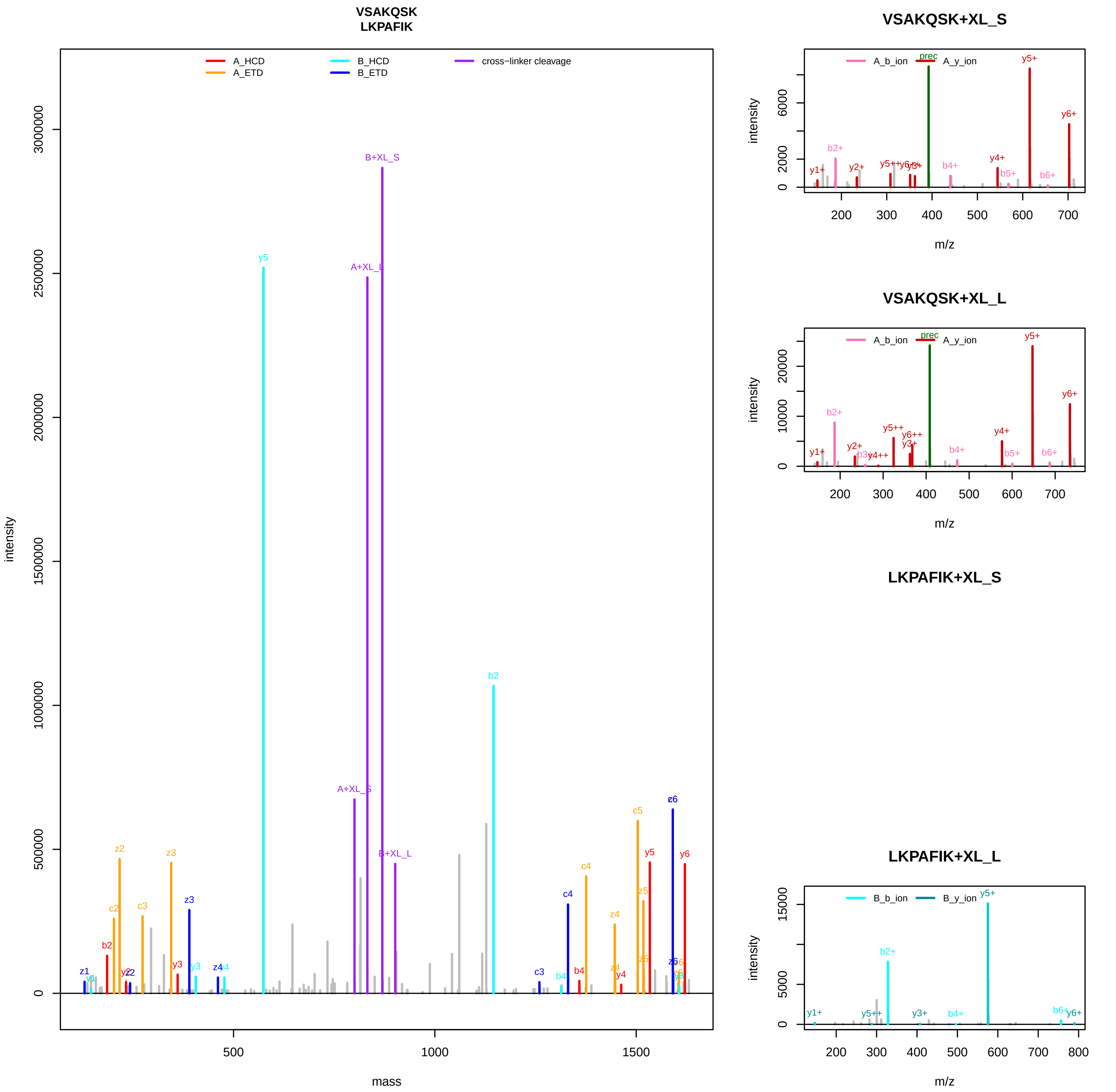
VSAKQSK
LKPAFIK
A_HCD
A_ETD
B_HCD
B_ETD
cross−linker cleavage
intensity
mass
0
500000
1000000
1500000
2000000
2500000
3000000
500
1000
1500
B+XL_S
A+XL_L
A+XL_S
B+XL_L
y5
b2
z1
b2
c2
z2
c3
z3
y3
z3
y3
z4
c3
b4
c4
b4
c4
z4
y4
c5
z5
y5
z6
y6
VSAKQSK+XL_S
A_b_ion
A_y_ion
prec
intensity
m/z
0
2000
6000
200
300
400
500
600
700
y1+
b2+
y2+
b4+
y4+
b5+
y5+
b6+
y6+
VSAKQSK+XL_L
A_b_ion
A_y_ion
prec
intensity
m/z
0
10000
20000
200
300
400
500
600
700
y1+
b2+
y2+
y5++
y6++
y3+
y4++
b4+
y4+
b5+
y5+
b6+
y6+
LKPAFIK+XL_S
LKPAFIK+XL_L
B_b_ion
B_y_ion
intensity
m/z
0
5000
15000
200
300
400
500
600
700
800
y1+
b2+
y5++
y3+
b4+
y5+
b6+
y6+
